# Severe Acute Respiratory Distress Syndrome in Lyme Disease: A Case Report and Review of the Literature

**DOI:** 10.7759/cureus.81170

**Published:** 2025-03-25

**Authors:** Chris Magloire, Tigran Aghabekyan, Nicholas E Morley, Sava Turcan, Shawn Sexton, Dilruba Khatoon, Chibuzo Okoye, Samy I. McFarlane

**Affiliations:** 1 Internal Medicine, State University of New York Downstate Health Sciences University, New York, USA; 2 Internal Medicine, State University of New York Downstate Medical Center, New York, USA; 3 Anesthesiology, State University of New York Downstate Health Sciences University, New York, USA; 4 Anesthesiology, State University of New York Downstate Medical Center, New York, USA; 5 Critical Care Medicine, State University of New York Downstate Health Sciences University, New York, USA

**Keywords:** acute respiratory distress syndrome (ards), ards, lyme's disease, lyme's disease and ards, tick-borne disease and ards

## Abstract

While there are many etiologies of acute respiratory distress syndrome (ARDS), Lyme disease is not a known cause of this disorder, and there is a paucity of Lyme-associated ARDS cases reported in the medical literature. In this report, we present a case of a 70-year-old woman with ARDS requiring mechanical ventilation, who initially had recurrent negative infectious workups but was ultimately diagnosed with Lyme disease with positive Lyme serology and western blot. The patient, who is from the New York City metropolitan area, had no outdoor exposure, recent travel history, or sick contacts. She experienced a complicated intensive care unit (ICU) course, including septic shock requiring antibiotics, vasopressors, and multiple diagnostic tests. Ultimately, the patient recovered and was transferred to the medicine unit and subsequently discharged in stable condition. This case highlights a rare complication of Lyme disease and highlights the importance of considering rare etiologies in the differential diagnosis of ARDS with an unclear etiology.

## Introduction

Acute respiratory distress syndrome (ARDS) is a severe form of respiratory failure that is characterized by non-cardiogenic pulmonary edema, hypoxemia, and characteristic bilateral pulmonary infiltrates on chest X-ray [1]. It is a multi-etiologic disease, with the most common cause being pulmonary infections, while pancreatitis, aspiration of gastric contents, and severe traumatic injuries with shock and multiple transfusions are other common causes [2]. Risk factors for ARDS include advanced age, female gender, smoking, and alcohol use [3]. Although tick-borne diseases have not been established as etiologic agents for ARDS, there have been rare reports of cases such as erichilosis, babesiosis, and Lyme disease associated with ARDS [4]. Most notably, a fatal case of ARDS was described in a case report of a patient with Lyme disease in 1988, suggesting a potential for pulmonary complications in patients with the disease [5]. In this paper, we present a case of ARDS in a patient with no clear infectious source, ultimately found to have Lyme disease despite no recent travel or high-risk exposure.

## Case presentation

A 70-year-old woman with a history of mild intermittent asthma, hypertension, obesity, fatty liver, and prediabetes presented to the emergency department with a one-week history of nausea, vomiting, and diarrhea. She also reported upper respiratory symptoms, including worsening shortness of breath, cough, and fever, for three days prior to admission. The patient also reported two falls without loss of consciousness one day prior to admission. She reports no chest pain, orthopnea, or lower extremity edema and no recent exacerbation of her asthma. She received the coronavirus disease 2019 (COVID-19) vaccine and two booster doses of the vaccine. In the emergency department (ED), the patient was tachypneic, with a respiratory rate of 30 and SpO2 of 90% on 2L of supplemental oxygen, but she was hemodynamically stable, with a heart rate of 79 bpm and blood pressure of 106/66 mm Hg. The calculated body mass index (BMI) (kg/m^2^) was 34.4 kg/m^2^, with a calculated body surface area (m^2^) of 2.1.

**Figure 1:**
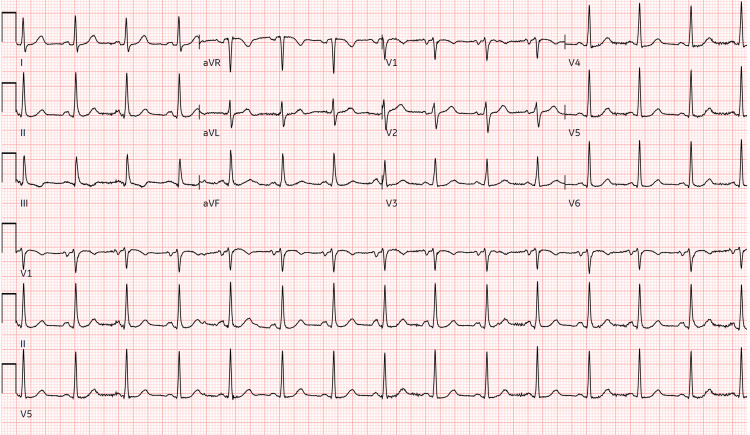
EKG of the patient on admission showing normal sinus rhythm

**Figure 2:**
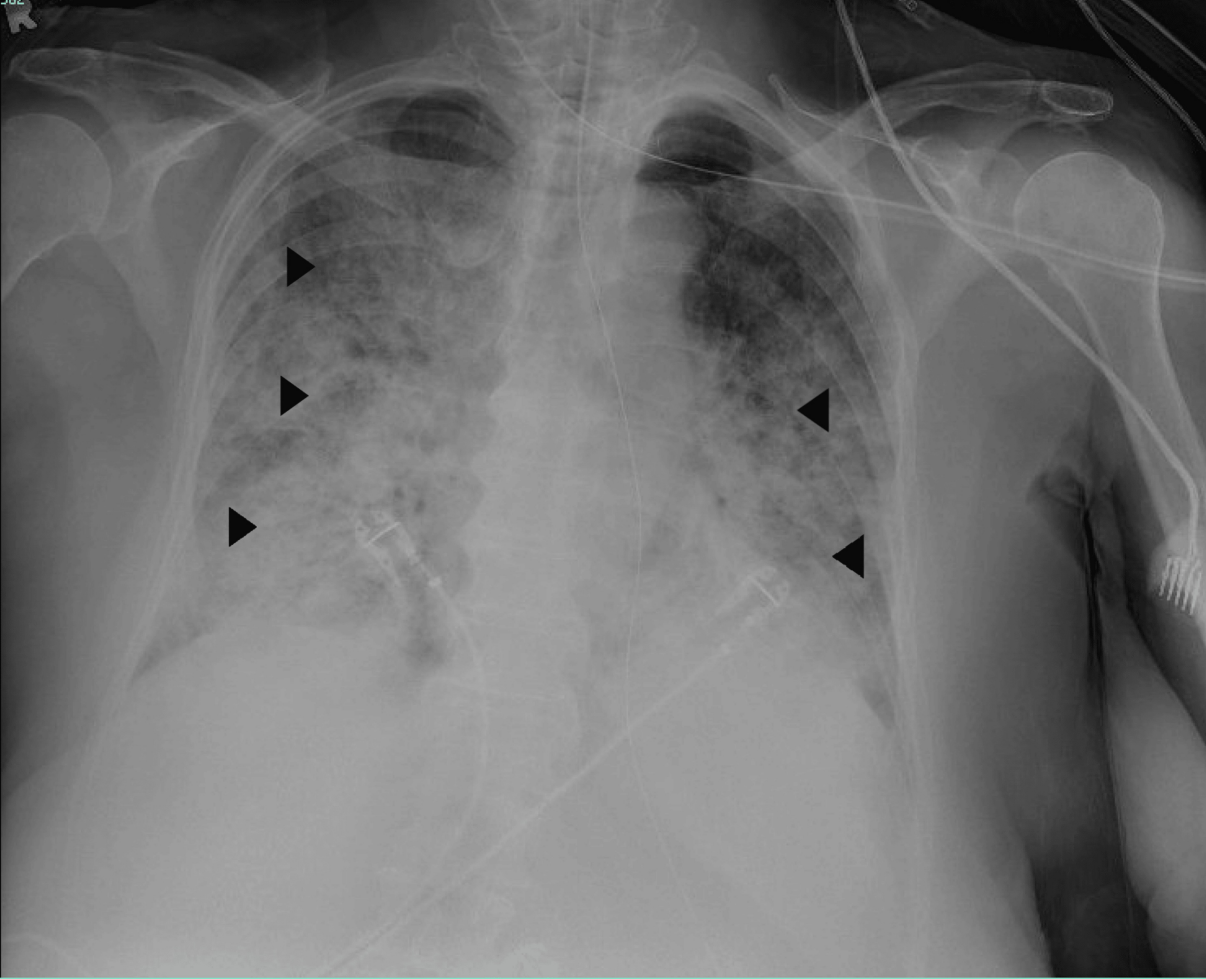
Chest X-ray revealing the presence of opacities (consolidations with air bronchograms) in multiple lung fields bilaterally (black arrowheads)

Physical examination was remarkable for labored breathing and moderate distress due to increased breathing work. Laboratory workup revealed mixed respiratory and metabolic acidosis (arterial pH 7.10, PaCO2 56.2, PaO2 84.6, HCO-3 16.7) and significant elevations in serum creatinine (3.0 mg/dL from normal baseline). Her brain natriuretic peptide was also elevated at 379 (normal values: < 100 pg/mL), with a negative high sensitivity troponin I and a white blood cell count of 10.2 (Table 1). The electrocardiogram (12-lead) was unremarkable and showed a normal sinus rhythm (Figure 1), and bedside point-of-care ultrasound did not raise suspicion about acute cardiac abnormalities. Well’s score for pulmonary embolism was zero at the time of admission, and further testing for PE was not considered. Chest X-ray demonstrated bilateral, predominantly peripheral, asymmetrical consolidation with air bronchograms (Figure 2). Given the presence of appropriate risk factors, findings of chest imaging, the timing of the respiratory failure being <7 days, and PaO2:FIO2 ≤100 mmHg, the patient was diagnosed with an initial diagnosis of ARDS based on the global definition of ARDS from 2024 [6].

**Table 1:** Laboratory investigations Na, Sodium; K, Potassium; Cl, Chloride; BUN, Blood Urea Nitrogen; WBC, White Blood Cell Count; AST, Aspartate Aminotransferase; ALT, Alanine Aminotransferase; ALP, Alkaline Phosphatase; TBili, Total Bilirubin

Lab test	Observed value	Reference range
Na	130 mEq/L	135 - 145 mEq/L
K	3.5 mEq/L	3.5 - 5.0 mEq/L
Cl	97 mEq/L	96 - 106 mEq/L
HCO₃⁻	17 mEq/L	22 - 26 mEq/L
Glucose	172 mg/dL	70 - 100 mg/dL (fasting)
Creatinine	3.0 mg/dL	0.6 - 1.2 mg/dL
BUN	51 mg/dL	7 - 20 mg/dL
Hemoglobin	9.9 g/dL	12 - 16 g/dL (female)
Hematocrit	29.50%	36% - 48% (female)
WBC	10,200 /μL	4,000 - 11,000/μL
Platelets	80,000 /μL	150,000 - 450,000/μL
Lactate	2.5 mmol/L	0.5 - 1.0 mmol/L
Arterial pH	7.1	7.35 - 7.45
PaCO₂	56.8 mmHg	35 - 45 mmHg
PaO₂	84.6 mmHg	75 - 100 mmHg
SaO₂	92%	95% - 100%
AST	110 U/L	10 - 40 U/L
ALT	69 U/L	7 - 56 U/L
ALP	216 U/L	40 - 130 U/L
Tbili	3.4 mg/dL	0.1 - 1.2 mg/dL

Due to worsening respiratory status and acidosis (venous blood pH from 7.31 to 7.22), a trial of non-invasive continuous positive pressure ventilation was initiated while the patient was still in the ED, with little benefit for the work of breathing and tachypnea. After goals of care discussions with the patient and her family, the patient was subsequently intubated, and invasive mechanical ventilation was initiated due to worsening respiratory distress. The patient also sustained an episode of hypotension with systolic blood pressure at 78 and heart rate at 130, one hour post-intubation, likely because of a self-resolving episode of atrial tachycardia. The patient was admitted to the medical intensive care unit (ICU) with the diagnosis of ARDS, presumably secondary to primary viral with superimposed bacterial infection complicated by acute kidney injury (AKI) and hypotension.

In the ICU, the patient was managed with a low tidal volume and lung-protective ventilation for ARDS [7]. Initial treatment also included empiric antibiotics (vancomycin and piperacillin-tazobactam) for presumed pulmonary septic shock. The patient was sedated with propofol, but on hospital day 3, the sedative medication was switched to midazolam due to the development of propofol-related infusion syndrome (triglyceride levels >800 mg/dL). Shortly after admission to the ICU, the patient developed hypotension secondary to sepsis and was started on a continuous norepinephrine infusion for a goal mean arterial pressure of more than 65 mm Hg. Norepinephrine was later switched to phenylephrine after the patient developed paroxysmal atrial fibrillation (AFib) with a rapid ventricular rate. The patient was successfully cardioverted and an antiarrhythmic medication (amiodarone) was initiated for the maintenance of sinus rhythm in the patient.

**Figure 3:**
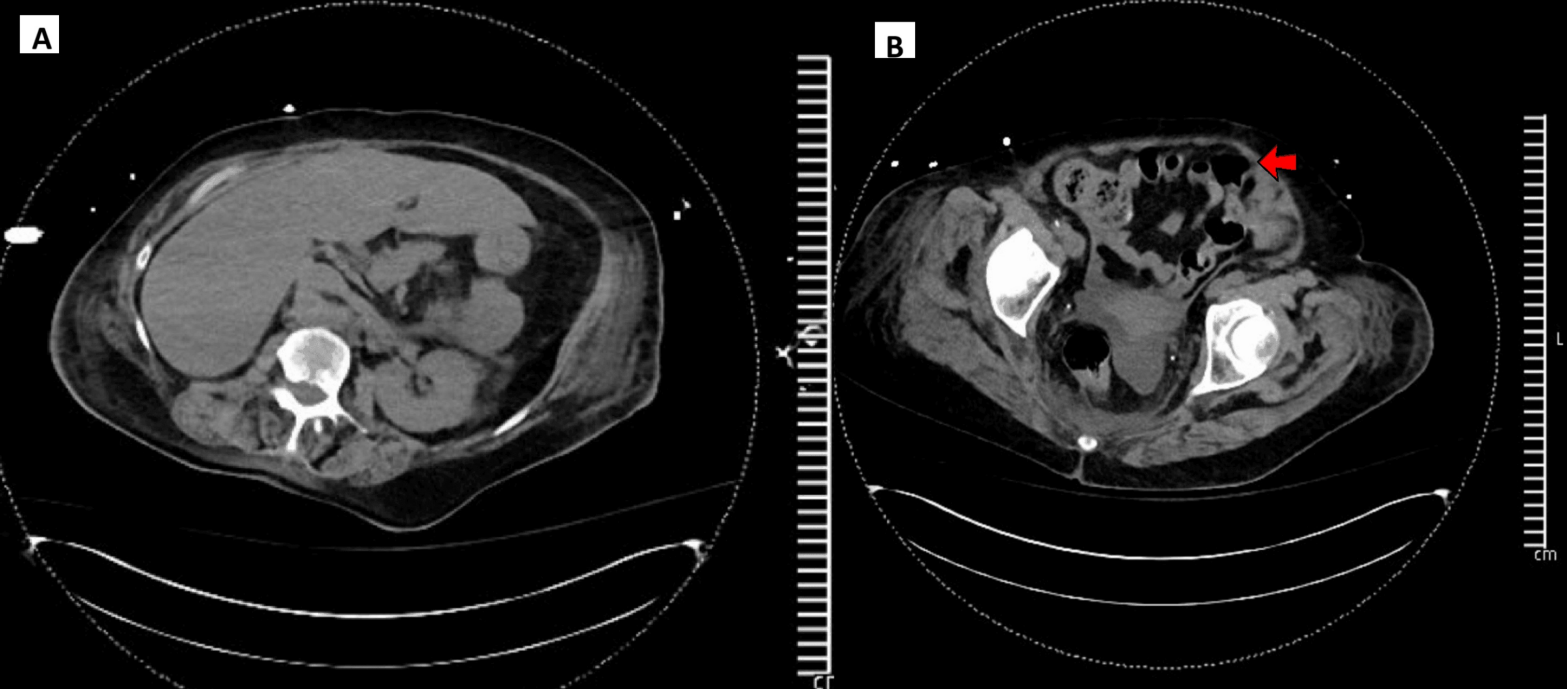
Cuts from the CT abdomen and pelvis demonstrating no sources of infections, only remarkable for diverticulosis (red arrow) without diverticulitis

An aggressive evaluation for a possible source of infection was started upon admission to the ICU. Initial testing included a 15-virus respiratory panel, two sets of blood cultures, urine culture, sputum culture, bronchoalveolar lavage analysis and culture, urinary pneumococcal and legionella antigen testing, lumbar puncture and cerebrospinal fluid analysis, and CT abdomen and pelvis (Table 2, Figure 3). These were all negative as possible sources of infection. The patient was continued on empiric vancomycin and piperacillin-tazobactam. The broad-spectrum antibiotics (i.e., vancomycin and piperacillin-tazobactam) were discontinued on hospital day 3 and levofloxacin was continued for empiric coverage for community-acquired pneumonia (with no risk factors for MRSA (negative nasal swab) or pseudomonas). Further infectious workup was directed by history and physical examination clues (tick bite, history of the characteristic rash and joint pain) revealed positive Lyme disease IgM/IgG titers on serology with reflex confirmation with Western Blotting. After extubation on hospital day 15, the patient confirmed that she had a rash and arthralgias about a year prior to admission, for which she never received treatment, and the symptoms spontaneously resolved. Treatment with doxycycline was initiated on hospital day 15, and on day 18, the antibiotic was switched to amoxicillin after the development of possible drug-induced eosinophilia (absolute eosinophil count at 3.48 with 24% of total white cells, timing consistent with the initiation of doxycycline).

**Table 2:** Workup of the patient for possible sources of infection

Test	Results
Blood culture, x2	No growth in five days
Urine culture	<10,000 CFU
Sputum culture	No growth
Respiratory viral panel	Not detected
Bronchoalveolar lavage culture	Few Candida albicans
Urinary pneumococcal antigen	Negative
Urinary legionella antigen	Negative
Legionella culture (sputum)	No organism identified
Cerebrospinal fluid culture	No growth
Lyme disease IgM/IgG immunoblotting	Positive

The ICU stay was complicated by the development of an upper gastrointestinal bleed (two episodes, on hospital days 5 and 10, approximately 200 mL and 50 mL of bright red blood, respectively). Upper endoscopy on hospital day 13 revealed mild, linear, non-bleeding erosions in the proximal body with granulation tissue in the cardia and esophagus, likely resulting from a combination of trauma secondary to the placement/presence of nasogastric tube as well as stress-related mucosal injury. Treatment with esomeprazole was initiated.

The patient was initiated on intermittent hemodialysis on hospital days 3 and 5 for persistent acute renal failure (initially prerenal based on calculated fractional excretion of sodium but further precipitating acute tubular necrosis) with an initial creatinine of 3.0 that increased to 4.8 despite fluid resuscitation and diuretic challenge. After three sessions of intermittent hemodialysis with an appropriate response in creatinine and blood urea nitrogen levels, treatment with furosemide and albumin was initiated. The AKI was resolved on hospital day 10.

She was slowly weaned off sedation and became alert, awake, and oriented on hospital day 13. She was extubated on hospital day 15 and transitioned to non-invasive ventilation with bilevel-positive airway pressures. Her respiratory status continued to improve, which resulted in her transition to nasal cannula oxygen therapy. On hospital day 19, the patient was successfully downgraded from the medical ICU to the general medical floor. She was discharged on hospital day 34 in stable condition with ICU-associated weakness but no focal neurological deficits.

## Discussion

ARDS is a common cause of respiratory failure in critically ill patients and is defined as an acute onset of noncardiogenic edema and hypoxemia, eventually requiring mechanical ventilation [1]. The Berlin criteria, developed in 2012 to standardize the diagnosis of ARDS, define it based on timing (within one week of a known insult or new worsening respiratory symptoms), origin of edema (not fully explained by cardiac failure or volume overload), imaging (bilateral opacities on chest x-ray or CT scan not fully explained by effusions, nodules or collapse, and a PaO2/FiO2 ratio less than 300 mmHg on at least 5 cmH_2_O of positive end-expiratory pressure [8]. The New Global Definition of ARDS, proposed in 2024, is built upon the Berlin criteria and offers a more comprehensive approach by adding high-flow nasal cannula requirements, SpO2, as well as lung ultrasound findings to the diagnostic criteria [6]. According to the LUNG-SAFE study, hospital mortality was 34.9% for patients with mild ARDS, 40% for moderate, and 46.1% for severe ARDS, although the study was unable to determine the contribution of comorbidities to the mortality rates [9]. The LUNG-SAFE study also revealed low ARDS recognition by clinicians: only 51% recognized ARDS in mild disease and 79% in severe disease. These high mortality and low recognition rates highlight the importance of clinicians understanding and identifying patients at risk who present with early signs of ARDS so treatment can be initiated early in the disease course.

Treatment of the underlying etiology is an essential part of therapy for ARDS [10]. However, multiple studies have highlighted the challenges of finding the specific underlying etiology in any case of ARDS, with one study documenting that about 24% of all cases eventually get labeled as ‘idiopathic’ and thereby preclude the etiology-specific treatment in addition to general ARDS management [11].

Lyme disease, caused by the spirochete Borrelia burgdorferi, is the most common vector-borne disease in the United States, primarily affecting individuals bitten by an Ixodes deer tick infected with the spirochete [12]. Lyme disease typically progresses through early, early disseminated, and late stages, with treatment usually halting further progression. The early stage presents a rash called erythema migrans, which lasts 14 to 21 days. The early disseminated stage can include multiple areas of erythema migrans, migratory arthralgias, carditis (presenting as a prolonged PR interval on EKG), and early neuroborreliosis (mild meningitis, isolated cranial nerve palsy, and radiculoneuropathy), which also lasts 14-21 days. The late stage involves arthritis, late neuroborreliosis, as well as a skin condition called acrodermatitis chronica atrophicans [13]. Despite the diverse manifestations of Lyme disease, a review of the literature suggests that respiratory symptoms are not well-established in Lyme disease and are usually limited to pharyngitis/cough [14]. Rarely, respiration can be compromised due to central hypoventilation caused by neurological complications of Lyme disease. There is only one case report describing a fatal instance of ARDS in a patient with Lyme disease [15]. The review of the published case revealed that the patient was a 67-year-old woman who initially presented with a rash on her extremities and trunk, cough, swelling of her hands, and fever-initially diagnosed as rocky mountain spotted fever. Subsequent testing revealed positive IgM and IgG titers for Lyme disease, and she completed an antibiotic course with tetracycline without improvement. She was then treated with a 10-day course of IV penicillin G, which resolved her fevers, joint pain, and elevated liver enzymes, leading to hospital discharge. Five days later, she was admitted with a fever of 100.6 (38.1 °C), a heart rate of 112 beats per minute, and respirations of 20 breaths per minute. During her stay, she developed rising fevers, worsening hypoxia on arterial blood gas analysis, and an increasing leukocytosis with bands. Her condition deteriorated, culminating in her death due to diffuse alveolar damage of the lungs. The authors suggested that her lack of response to standard treatments like tetracycline and high-dose IV penicillin might have been due to a particularly virulent spirochete or a disease stage no longer reliant on the live spirochetes. This case highlights the importance of early and accurate identification of the pathogen responsible for successful ARDS treatment. Fortunately, our patient was diagnosed with Lyme disease within nine days and treated appropriately, which may have contributed to her favorable outcome.

Although rare, the possibility of respiratory manifestations shouldn’t be overlooked in patients with Lyme disease, and conversely, the tick-borne illness should be considered in patients with ARDS.

## Conclusions

We report a rare case of ARDS associated with Lyme disease that was ultimately considered after an extensive negative infectious workup was pursued, yielding a timely the diagnosis and favorable outcomes. Our report highlights the importance of considering tick-borne disease in the differential diagnosis of ARDS with careful history taking, physical examination, and diagnostic workup to institute specific therapy for better clinical outcomes.
